# Assessing the impact of combined nutritional and physical activity interventions for patients with atrial fibrillation who are overweight: a systematic review protocol

**DOI:** 10.1136/bmjopen-2026-119196

**Published:** 2026-06-23

**Authors:** David Murphy, John Graby, Lisa Hirst, Jonathan Rodrigues, Dylan Thompson, Ali Khavandi

**Affiliations:** 1 Department of Health, University of Bath, Bath, UK; 2 Royal United Hospitals Bath NHS Foundation Trust, Bath, UK

**Keywords:** CARDIOLOGY, Body Mass Index, REHABILITATION MEDICINE

## Abstract

**Abstract:**

**Introduction:**

Atrial fibrillation (AF) is the most common sustained cardiac arrhythmia and is associated with reduced quality of life and increased healthcare utilisation. AF is increasingly recognised as a manifestation of an underlying cardiometabolic disease process, with obesity and related metabolic risk factors contributing to its development and progression. Consequently, upstream risk factor modification, including structured weight management, has emerged as a potential strategy to improve AF outcomes. While dietary and exercise interventions have been studied individually, the effectiveness of programmes combining both components has not been systematically reviewed in patients with AF who are overweight or obese. This review aims to assess the impact of combined nutritional and physical activity interventions on AF-related outcomes.

**Methods and analysis:**

A systematic search will be conducted in MEDLINE, CINAHL, EMBASE and EMCARE from database inception to April 2026. Eligible studies will include randomised controlled trials, quasi-experimental studies and controlled cohort studies evaluating combined dietary and physical activity interventions in adults with AF who are overweight or obese. The primary outcome will be AF symptom burden measured using validated instruments. Secondary outcomes will include quality of life, anthropometric measures, metabolic parameters and AF arrhythmia burden. Risk of bias will be assessed using RoB 2 and Risk of Bias in Non-randomised Studies of Interventions. Where appropriate, meta-analysis will be performed using random-effects models.

**Ethics and dissemination:**

Ethical approval is not required. Findings will be disseminated through peer-reviewed publications and scientific meetings.

**PROSPERO registration number:**

Open Science Framework (OSF; https://doi.org/10.17605/OSF.IO/HCUZ3).

**STRENGTHS AND LIMITATIONS OF THIS STUDY**

- A comprehensive multi-database search, with reference screening, will minimise the risk of missing relevant studies.
- Prospective registration with prespecified criteria enhances transparency and reduces selective reporting.
- Independent screening, data extraction and risk of bias assessment using validated tools reduces error and subjective bias.
- Heterogeneity in interventions and outcome measures may limit comparability and meta-analysis.
- Inclusion of non-randomised studies and restrictive criteria may introduce confounding and limit study numbers.

## Introduction

### Background

 Atrial fibrillation (AF) currently affects approximately 1.5 million people in the United Kingdom (UK), leading to impaired health-related quality of life and a reduced life expectancy.^1^,^3^ It is also a marker of increased risk for other cardiovascular and systemic conditions, including heart failure, stroke and cognitive decline.^4^,^6^ Both the prevalence and incidence of AF are increasing, with projections suggesting that nearly 3 million people in the UK will be living with AF by 2040. Correspondingly, the direct costs to the National Health Service (NHS) are estimated to comprise around 4% of the total NHS budget.^7 8^

Traditional management of AF centres on the use of anti-arrhythmic and rate-controlling medications. However, these pharmacological approaches have limited efficacy in maintaining sinus rhythm.^9^ Other treatment modalities, such as direct current cardioversion and AF catheter ablation procedures, play an important role, especially in some patient subsets, but are not without limitations.^10 11^ These procedures require a hospital admission, general anaesthesia and carry a risk of complications.^12 13^ Furthermore, the effectiveness of these procedures tends to decline with time, with a significant rate of AF recurrence.^14 15^

The high recurrence rate of AF is frequently attributed to the ongoing changes to the underlying myocardium, including atrial dilatation, fibrosis and impaired electrical conduction.^16^ AF is increasingly recognised not merely as a stand-alone arrhythmia but as a clinical manifestation of an atrial cardiomyopathy, driven by cardiometabolic risk factors such as arterial hypertension, hyperglycaemia and dyslipidaemia.^17 18^ This paradigm has shifted attention towards upstream risk-factor modification as a potential strategy to alter the natural history of the disease.

Elevated body mass index (BMI) often serves as a driver of many of these risk factors, and therefore the targeted reduction may exert pleiotropic benefits across several mechanistic pathways simultaneously. In recognition of these associations, several studies have suggested that structured weight loss programmes can improve AF symptoms and burden.^19^,^22^ Weight loss has been linked to dose-dependent improvements in long-term sinus rhythm maintenance, alongside beneficial effects on cardiometabolic profiles and favourable cardiac remodelling.^19^

While international guidelines now recommend addressing modifiable risk factors in all patients with AF,^9^ no established programme exists in current UK clinical pathways. Moreover, the optimal structure of such an intervention remains unclear. Prior reviews have evaluated nutritional or physical activity interventions in isolation, but evidence on combined lifestyle interventions incorporating both dietary modification and physical activity in patients with AF is lacking.^23^,^25^ This is despite combined interventions being more effective for achieving and sustaining clinically meaningful weight loss.^26^

This systematic review and meta-analysis therefore aim to evaluate the effects of combined nutritional and physical activity interventions on AF symptom burden, health-related quality of life, anthropometric measurements, metabolic markers and AF arrhythmia burden in patients with AF who are overweight or obese. In doing so, it may identify key features of the most effective interventions. Reducing symptom burden may decrease reliance on invasive treatments, such as catheter ablation, while potentially improving the durability of rhythm control by addressing the underlying metabolic drivers of AF.

## Specific objectives

Primary:

1. To identify and summarise studies that assess the effects of combined nutritional and physical activity interventions on patients with AF who are overweight or obese.
2. To quantify the relative effect of these interventions on AF symptom burden. Any validated AF symptom burden questionnaires or classification was eligible for inclusion.

Secondary:

1. To identify and quantify any effects of these interventions on:
  1. Anthropometric measurements (eg, body weight, BMI, waist circumference).
  2. Metabolic parameters (eg, lipid profile).
  3. AF arrhythmia burden (eg, recurrence rate).

## Criteria for study selection

Following the PICOS framework (Population, Intervention, Comparator, Outcomes, Setting, Study design) studies will be included based on the following criteria:

*Population:* adults with existing AF (paroxysmal, persistent or permanent) who meet the criteria for being overweight or obese, according to standard criteria (BMI ≥25 kg/m^2^).

*Intervention:* any combined nutritional and physical activity intervention that has a clear description of the nature, duration and specific parameters used. Combined interventions have been defined as programmes incorporating both a structured dietary component and a structured physical activity component delivered as part of a single intervention.

*Control/comparison:* studies comparing the combined intervention to either (a) A nutritional or physical activity intervention administered in isolation or (b) A control group receiving no intervention, provided that all interventions are clearly described with respect to their content and delivery parameters. Where sufficient data are available, analyses will be stratified according to comparator type (combined intervention vs single-component intervention, and combined intervention vs usual care) to minimise clinical heterogeneity.

*Outcomes of interest:* primary outcome: AF symptom burden (any validated questionnaires will be included). This was selected as it is a clinically relevant, patient-centred outcome that informs management decisions in routine practice. Secondary outcomes: health-related quality of life measurements, anthropometric measurements (eg, weight, waist circumference and BMI), metabolic markers (eg, HbA1C, HDL-C, LDL-C and glucose), physical fitness (eg, VO_2_ peak, 6 min walk test), AF burden (eg, data from implantable cardiac monitors, Holter monitors).

*Setting:* initiation of any intervention will be within a healthcare setting, but the delivery could be within a community setting (eg, community gym, at home).

*Study designs:* randomised controlled trials (RCTs), quasi-experimental studies and prospective or retrospective controlled cohort studies will be eligible. As the systematic review seeks to examine effectiveness outcomes, single-arm studies, case reports or series and feasibility studies will also be omitted.

## Criteria for exclusion

1. Previous ablation: studies involving patients who have explicitly undergone a previous ablation procedure will be excluded. Studies focusing on the initial point of referral to an electrophysiology service will only be included where previous ablation has not been reported or where the majority of patients were ablation naïve. Patients who have undergone prior ablation often represent a subgroup with more persistent or treatment-resistant AF, frequently referred following failure of medical therapy. Modification of cardiometabolic risk factors is hypothesised to influence AF primarily through reversal of atrial remodelling, mechanisms that are likely to be most effective earlier in the disease course. The review aims to focus on earlier stages of the disease trajectory, where such modification may have the greatest potential.
2. Unclear intervention description: studies where the description of the intervention lacks specific detail will be excluded.
3. No comparator group: studies without a comparator group (eg, single-arm cohort studies stratifying participants post hoc based on outcome) will be excluded.
4. Short follow-up duration: studies with less than 6 months of post-intervention follow-up will be excluded.

## Methods and analysis (research plan)

### Data search methods

This systematic review protocol was prospectively registered on the Open Science Framework (OSF; https://doi.org/10.17605/OSF.IO/HCUZ3).

Relevant literature will be systematically searched in the following databases: Medline, CINAHL, EMBASE and EMCARE from database inception to April 2026.

All identified articles will be exported into Covidence for screening and data extraction. In addition to the database search, the reference list of all studies undergoing data extraction will be screened for further relevant publications, as will any relevant systematic reviews or review papers highlighted via the search. Detailed search criteria for Medline are provided in online supplemental appendix A, and will be adapted for other databases. The strategy was developed by the review team, tailored to the key index terms identified in the preliminary results and reviewed for relevance and appropriateness by a hospital librarian.

Studies using datasets that substantially overlap with other included studies will be excluded to avoid duplication of data. In cases where multiple studies report on the same cohort or database, the most comprehensive or recent publication will be included.

Any important protocol amendments will be documented and dated within the OSF registration and clearly reported in the final systematic review publication.

## Methods of review

Two main reviewers (JG and DM) will independently screen titles and abstracts of the search results for relevance and eligibility based on the agreed inclusion and exclusion criteria. Any disagreements will be resolved by a third reviewer (AK).

The full text of the remaining studies will then be assessed for data extraction. Data extraction will be conducted using Covidence, and all information will be recorded and managed both digitally and manually. The data extraction will capture the following key fields: study title; authors; year of publication; study size and design; details of type of intervention used, including description of intervention parameters (eg, type of diet or exercise and duration of intervention); changes in key study metrics of interest, for example, quality of life/symptom scores. Relevant comorbidities, including heart failure where reported, will also be recorded. Following extraction, this data will then be exported as a summary table to Excel for further analysis. Reasons for exclusion will also be recorded in the review process.

## Quality assessment (risk of bias)

Assessment of study quality and risk of bias will be conducted using the second version of the Cochrane Risk of Bias tool (RoB 2) for RCTs.^27^ This tool evaluates bias across key domains including randomisation process, deviations from intended interventions, missing outcome data, measurement of outcomes and selection of the reported result. For non-randomised (observational) studies, risk of bias will be assessed using the ROBINS-I (Risk Of Bias In Non-randomised Studies of Interventions) tool.^28^ ROBINS-I evaluates bias across seven domains: bias due to confounding, selection of participants, classification of interventions, deviations from intended interventions, missing data, measurement of outcomes and bias in the selection of the reported results.

Risk of bias will be assessed independently by the two main reviewers (DM and JG) and rated as having low, moderate or high risk of bias, or no information, leading to an overall risk of bias rating for each study. Any disagreements are resolved first by discussion and then by the input of a third reviewer (AK) if necessary.

## Data synthesis

The study selection process will be summarised using a Preferred Reporting Items for Systematic Reviews and Meta-Analyses (PRISMA)-recommended study flow diagram. A systematic narrative synthesis will be produced using both textual descriptions and tabular summaries. This synthesis will compare the results across studies and draw conclusions based on the evidence.

For each study outcome, a meta-analysis will be conducted when at least two studies report relevant data. For continuous outcomes, mean differences will be used when outcomes were measured using the same scale. Where studies report continuous outcomes as medians and IQRs these values will be converted to approximate means and SDs using the method described by Wan *et al*.^29^ This approach estimates the mean and SD from median, IQR and sample size, allowing for more consistent pooling of data across studies.

In studies reporting change scores without providing the correlation between baseline and follow-up measures, a default correlation coefficient of *r*=0.5 will be assumed, following recommendations from the Cochrane Handbook for Systematic Reviews of Interventions.^30^

Given the anticipated clinical and methodological heterogeneity among studies, random-effects models will be chosen a priori for all meta-analyses, using the DerSimonian and Laird method.^31^ Statistical heterogeneity will be assessed and quantified using both τ² (between-study variance) and I² statistics.

If at least 10 studies are available for a given outcome, publication bias will be assessed using funnel plots. Where fewer than 10 studies are available, quantitative assessment of publication bias will not be undertaken due to low statistical power and the risk of spurious asymmetry; instead, the potential for publication bias will be discussed qualitatively.

Where appropriate, the certainty of evidence for outcomes will be assessed using the Grading of Recommendations Assessment, Development and Evaluation framework. This approach evaluates certainty across domains including risk of bias, inconsistency, indirectness, imprecision and publication bias.

### Study timeline

At the time of protocol submission, the literature search had been started, but screening and data extraction had not yet commenced.

### Patient and public involvement

Patients and members of the public were not involved in the design of this systematic review protocol and will not be involved in data collection, extraction and analysis.

### Ethics and dissemination

Ethical approval is not required for this systematic review as it will involve analysis of previously published data and will not include individual patient data. The findings of this review will be disseminated through publication in a peer-reviewed journal.

## Supplementary material

10.1136/bmjopen-2026-119196 online supplemental file 1

## Data Availability

No data are available.
