# Supplementary material for: Assessing the impact of combined nutritional and physical activity interventions for patients with atrial fibrillation who are overweight: a systematic review protocol
Source: BMJ Open. 2026 Jun 23;16(6):e119196. doi: 10.1136/bmjopen-2026-119196 (PMC13295765; doi:10.1136/bmjopen-2026-119196)
Supplement: online supplemental file 1 [file bmjopen-16-6-s001.docx]

**Appendix A: Search strategies**

1. **Medline search strategy**

1 *Atrial Fibrillation/

2 atrial fibrillation*.ti.

3 AF.ti.

4 AFib.ti.

5 heart arrhythmia*.ti.

6 cardiac arrhythmia*.ti.

7 1 or 2 or 3 or 4 or 5 or 6

8 exp Obesity/

9 obes*.mp.

10 overweight.mp.

11 over weight.mp.

12 exp Overweight/

13 body mass.mp.

14 BMI.mp.

15 lipid*.mp.

16 risk factor*.mp.

17 lifestyle*.mp.

18 life style*.mp.

19 exp Body Mass Index/

20 exp Body Weight/

21 body weight.mp.

22 exp Risk Factors/

23 exp Life Style/

24 underlying condition*.mp.

25 weight.mp.

26 8 or 9 or 10 or 11 or 12 or 13 or 14 or 15 or 16 or 17 or 18 or 19 or 20 or 21 or 23 or 24 or 25

27 lifestyle chang*.ti,ab.

28 life style chang*.ti,ab.

29 lifestyle intervention*.ti,ab.

30 life style intervention*.ti,ab.

31 lifestyle modification*.ti,ab.

32 life style modification*.ti,ab.

33 behavio?r modification*.ti,ab.

34 behavio?r chang*.ti,ab.

35 intervention*.ti,ab.

36 weight manag*.ti,ab.

37 weight loss*.ti,ab.

38 secondary prevention*.ti,ab.

39 exp Secondary Prevention/

40 exp Weight Reduction Programs/

41 nutrition*.ti,ab.

42 diet*.ti,ab.

43 exp Nutrition Therapy/

44 physical activit*.ti,ab.

45 exercis*.ti,ab.

46 aerobic*.ti,ab.

47 anaerobic*.ti,ab.

48 exp Exercise Therapy/

49 weight reduc*.ti,ab.

50 exp Weight Loss/

51 risk factor management.ti,ab.

52 risk factor reduction*.ti,ab.

53 risk factor modification*.ti,ab.

54 risk reduction behavio?r*.ti,ab.

55 exp Risk Reduction Behavior/

56 exp Diet Therapy/

57 (reduc* adj3 body mass).ti,ab.

58 (reduc* adj3 BMI).ti,ab.

59 (reduc* adj3 weight).ti,ab.

60 rfm.ti,ab.

61 27 or 28 or 29 or 30 or 31 or 32 or 33 or 34 or 35 or 36 or 37 or 38 or 39 or 40 or 41 or 42 or 43 or 44 or 45 or 46 or 47 or 48 or 49 or 50 or 51 or 52 or 53 or 54 or 55 or 56 or 57 or 58 or 59 or 60

62 exp ANIMALS/ not exp HUMANS/

63 7 and 26 and 61

64 63 not 62
