# Supplementary material for: Assessing the impact of combined nutritional and physical activity interventions for patients with atrial fibrillation who are overweight: a systematic review protocol
Source: BMJ Open. 2026 Jun 23;16(6):e119196. doi: 10.1136/bmjopen-2026-119196 (PMC13295765; doi:10.1136/bmjopen-2026-119196)
Supplement: Supplementary file 2 [file bmjopen-2026-119196.reviewer_comments.pdf]

PEER REVIEW HISTORY

BMJ Open publishes all reviews undertaken for accepted manuscripts. Reviewers are asked to complete a checklist review form and are provided with free text boxes to elaborate on their assessment. These free text comments are reproduced below.

ARTICLE DETAILS

Title (Provisional)

Assessing the impact of combined nutritional and physical activity interventions for patients with atrial fibrillation who are overweight: a systematic review protocol

Authors

Murphy, David; Graby, John; Hirst, Lisa; Rodrigues, Jonathan Carl Luis; Thompson, Dylan; Khavandi, Ali

---

VERSION 1 - REVIEW

**Reviewer**	**1**
**Name**	**Lanza, Gaetano**
**Affiliation**	**Catholic University of the Sacred Heart**
**Date**	**04-Apr-2026**
**COI**	**None**

In this manuscript the authors report a study protocol aimed to assess the effects of combined nutritional and physicl activity interventions in overweight patients with atrial fibrillation (AF).

1. The authors state that dietary and exercise interventions have been studied individually, but not in combination in overweight/obese patients (page 2, lines 33-36).

However, at page 6, lines 159-162, they state that studies will be selected including “any combine nutritional and physical activity intervention”, which seems in contrast with the previous statement.

2. It should be specified if patients with various types of AF (paroxysmal, persistent, permanent) will be included.

2. The authors state that the primary end-point of the study will be AF symptom burden. Although standardized questionnaire exist on the topic, this reviewer is very puzzled with the choice of this rather generic outcome as the primary end-point. Symptom burden might indeed be significantly influenced by the effects of weight reduction on symptoms independent of AF. The choice of a more objective and strictly AF-related end-point (e.g.,

recurrences of AF episodes, heart rate during AF, possibly during Holter monitoring, use and number of antiarrhythmics, interventional procedures, etc…) would be desirable.

**Reviewer**	**2**
**Name**	**Zagidullin, Naufal**
**Affiliation**	**Bashkir State Medical University, Internal Diseases**
**Date**	**05-Apr-2026**
**COI**	**None**

This review aims to assess the impact of combined nutritional and

physical activity interventions on AF related outcomes in systhematic review.

It is well written and the idea is interesting to time and done according to the PRISMA protocol. There are some issues that could be discussed before publishing:

1) Please present the design of flow-chat Fig,

2) Please indicate also the type AF (patoxysmal, permanent, etc.) in the review.

3) Also indicate the existaнnce of heart failure as an inclusion criteria which may lead to the increase of body weight and decrease of physical activity.

VERSION 1 - AUTHOR RESPONSE

Reviewer 1:

1. The authors state that dietary and exercise interventions have been studied individually, but not in combination in overweight/obese patients (page 2, lines 33-36). However, at page 6, lines 159-162, they state that studies will be selected including "any combine nutritional and physical activity intervention", which seems in contrast with the previous statement.

Thank you for highlighting this inconsistency. We have revised the wording to clarify that, although dietary and physical activity interventions have been studied individually, evidence on their combined use in patients with atrial fibrillation and overweight or obesity remains limited and has not been systematically synthesised.

2. It should be specified if patients with various types of AF (paroxysmal, persistent, permanent) will be included.

Thank you for this suggestion. We have revised the eligibility criteria to specify that studies including patients with any type of AF (paroxysmal, persistent or permanent) will be eligible for inclusion, where reported.

3. The authors state that the primary end-point of the study will be AF symptom burden. Although standardized questionnaire exist on the topic, this reviewer is very puzzled with the choice of this rather generic outcome as the primary end-point.

Symptom burden might indeed be significantly influenced by the effects of weight reduction on symptoms independent of AF. The choice of a more objective and strictly AF-related end-point (e.g., recurrences of AF episodes, heart rate during AF, possibly during Holter monitoring, use and number of antiarrhythmics, interventional procedures, etc…) would be desirable.

Thank you for this important comment. We acknowledge that symptom burden may be influenced by factors beyond AF alone. However, we selected AF symptom burden as the primary outcome because it represents a clinically meaningful and patient-centred endpoint that directly informs treatment decisions in routine practice. In patients with AF, symptom severity is a key determinant of management strategies, including the use of antiarrhythmic therapy and referral for interventional procedures.

In addition, there is considerable heterogeneity in the reporting of objective AF-related outcomes (e.g. AF recurrence, AF burden on monitoring, heart rate control, or antiarrhythmic use) across studies, which may limit their suitability as a single primary outcome in a systematic review. These outcomes will therefore be included as secondary outcomes where reported, allowing a more comprehensive assessment of intervention effects.

We have clarified this rationale in the manuscript.

Reviewer: 2

1. Please present the design of flow-chat Fig,

Thank you for this suggestion. As this is a protocol, a study selection flow diagram cannot yet be presented. However, the study selection process will be reported using a PRISMA flow diagram in the completed systematic review.

2. Please indicate also the type AF (patoxysmal, permanent, etc.) in the review.

Thank you we have added this.

2. Also indicate the existaнnce of heart failure as an inclusion criteria which may lead to the increase of body weight and decrease of physical activity.

Thank you for this important comment. We agree that heart failure may influence body weight and physical activity levels. As such, we have clarified that the presence of heart failure will be recorded where reported and considered in the interpretation of findings.
